# Green and Efficient Ultrasonic-Assisted Extraction of Bioactive Components from *Salvia miltiorrhiza* by Natural Deep Eutectic Solvents

**DOI:** 10.3390/molecules25010140

**Published:** 2019-12-29

**Authors:** Xinping He, Jiehong Yang, Yan Huang, Yin Zhang, Haitong Wan, Chang Li

**Affiliations:** Zhejiang Chines Medical University, Hangzhou 310053, Chinayjhong@zcmu.edu.cn (J.Y.); 17081552478@163.com (Y.Z.)

**Keywords:** green extraction, natural deep eutectic solvent, *Salvia miltiorrhiza*, ultrasonic assisted extraction

## Abstract

Natural deep eutectic solvents (NaDESs) are recently developed green solvent alternatives to conventional fossil solvents. The present work systematically screened 22 different NaDESs for the ultrasonic-assisted extraction of bioactive components from *Salvia miltiorrhiza* (SM), a widely used traditional Chinese medical plant. The suitable solvent and extraction condition were optimized in a two-round screening. In comparison with fossil solvents, NaDESs, especially L-proline-lactic acid (L-Pro-Lac) showed significant advantages in the extraction of salvianolic acid B (SAB), tanshinone IIA (TIIA) and cryptotanshinone (CYT). The optimized yields of the three targeting compounds were 42.05, 1.485 and 0.839 mg/g, respectively. The present method was also applied to the pretreatment of SM samples from different geographic origins. The 2,2-diphenyl-1-picrylhydrazyl (DPPH) radical scavenging activities of NaDES extracts were determined in the study to prove the feasibility of NaDES in bioactive component extraction. The application of NaDESs in the extraction of both hydrophilic and hydrophobic small molecules from SM is proved to be a green and efficient method for pretreatment of herbal materials.

## 1. Introduction

The development of novel green solvent alternatives to conventional fossil solvents has long been considered as one of the major goals of sustainable chemistry. Greener solvents may alleviate environmental pollution and risks to human health as well. In recent decades, natural deep eutectic solvents (NaDESs) have gradually attracted the interest and attention from both scientific and industry areas [1]. NaDES is a eutectic mixture at room temperature formed from naturally occurring hydrogen bond donors (HBD) and hydrogen bond acceptors (HBA) [2], which can interact with each other through hydrogen bonds. As a special type of ionic liquid (IL), NaDES shares some similar properties to IL, such as negligible volatility, easy preparation, high dissolving capacity, conductivity and designability. In addition, NaDESs have got some advanced features, including better biodegradability, lower toxicity, 100% atom economy in preparation, and better biocompatibility. 

Due to the interesting physicochemical properties of NaDESs, their applications have been intensively investigated in various areas, ranging from electrochemistry, nanotechnology, catalysis and biomedical researches. NaDESs have also been applied to the extraction of various natural bioactive compounds [3,4], such as phenols [5], flavonoids [6,7], terpenoids [8], sugars [9], and alkaloids [10], etc. In a pioneering work by Dai et. al. [11], both polar and non-polar phenolic metabolites from *Carthamus tinctorius* L. were successfully extracted in high yields using three different NaDESs. Liu group [10] evaluated the extraction capacities of 43 types of NaDESs for alkaloids, flavonoids, saponins, anthraquinones, and phenolic acids from five Chinese herbal medicines. Very recently, Su group [12] developed a two-phase extraction system, consisting of hydrophilic and hydrophobic DESs, to extract flavonoids, terpene trilactones, procyanidines, and polyprenyl acetates from *Ginkgo biloba* leave. The aforementioned researches indicated that optimized DES systems showed high efficiency and convenience for different types of compounds covering a wide range of polarity.

**Figure 1:**
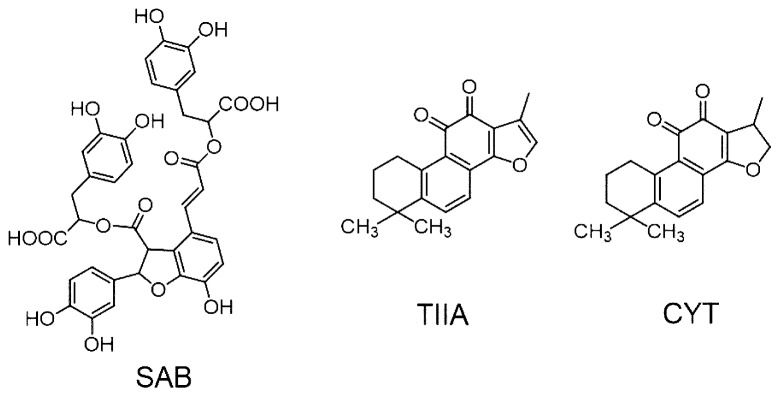
Chemical structures of SAB, TIIA and CYT.

*Salvia miltiorrhiza* (SM, Danshen in Chinese), which is one of the most popular herbal plants in East Asia, has long been widely used in clinical practices and phytomedicine researches [13,14]. According to Chinese Pharmacopoeia (2015 Ed.), there are 146 herbal medical products consisting of *Salvia miltiorrhiza* in the Chinese market. In clinic, SM has been intensively applied to the treatment of cardiovascular and cerebrovascular diseases in China, Japan and the United States [15,16]. The major components of SM, phenolic acids and tanshinones (Figure 1) have been proved important in its bioactivities and clinical effects. Phenolic compounds, including salvianolic acid B (SAB), danshensu and protocatechualdehyde, are the major water-soluble components in the plant. Scientific investigations have revealed that they have beneficial bioactivities as antioxidant [17], anti-blood coagulation [18] and neuroprotection [19]. Tanshinones, especially tanshinone IIA (TIIA) and cryptotanshinone (CYT), were also reported to have anti-cancer [20], anti-inflammatory hepatoprotective [21], and cardioprotective effects [22].

Thus, it is of great importance to develop an effective process for the extraction of the active components, polyphenols and tanshinones from *Salvia miltiorrhiza*. Most researches used routine solvents, such as water, methanol and ethanol, in either reflux or room temperature conditions. It has been reported that traditional extraction solvents showed inevitable drawbacks including long extraction time, huge solvent consumption and low utilization of the plant material. In Chinese Pharmacopoeia (2015 Ed.), water is the suggested solvent for the extraction of SAB, while ethyl ether is used for TIIA. Chen et. al. [23] reported a DES (ChCl as HBA)-based microwave-assisted extraction (MAE) method to extract SAB and TIIA together with rosmarinic acid, lithospermic acid and salvianolic acid A. The extraction efficiency was significantly improved in a solid/liquid (S/L) ratio at 5 mg/mL. 

Herein, we reported an improved, green and efficient extraction method for the components in *Salvia miltiorrhiza* based on NaDESs, where ultrasonic-assisted extraction (UAE) was used for its lower energy consumption and more concise operation. In total, 22 different NaDESs were prepared and the extraction effects were comprehensively studied. The extraction factors including S/L ratio, temperature, ultrasonic power, time, and water content, were systematically investigated. The established method was proved to be of higher efficiency, lower energy consumption and concise operability. The application of the optimal NaDES in the extraction of *Salvia miltiorrhiza* from different origins was also carried out. Furthermore, DPPH radical scavenging model was applied to evaluate the bioactivities of NaDES extracts to guarantee the activity maintenance of the extraction process.

## 2. Results

### 2.1. Screening of NaDESs for the UAE Effect on SM

**Figure 2:**
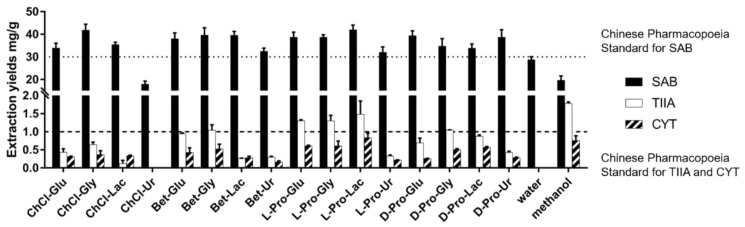
Bioactive components extraction yields from SM using NaDESs and traditional solvents (the first-round screening, under the initial conditions).

As presented in Table 1, four HBAs were selected, namely choline chloride, betaine, D-proline, and L-proline in the initial screening of NaDESs. At the same time, glucose, glycerol, lactic acid, and urea were used as HBDs, considering that they might represent four different types of routine HBDs, saccharides, alcohols, acids, and alkaloids, respectively. The molar ratios of HBA/HBD and water contents were applied according to previous reports [6,10]. In order to reveal the effect of NaDESs individually, initial fixed extraction factors were set as follows, S/L ratio at 100 mg/mL, DES content at 75%, extraction temperature at 50 °C, and an extraction time of 30 min. The results are summarized in Figure 2 (for detailed data, see Table S2 in Supporting Information).

Among the tested traditional solvents, water exhibited good extraction efficiency to SAB (28.78 mg/g), but TIIA and CYT were not detected in aqueous extract of SM. On the contrary, methanol extracted TIIA (1.788 mg/g) and CYT (0.761 mg/g) well, but SAB (19.67 mg/g) was in relatively low yield. It is noteworthy that most NaDESs showed increased capacities in the extraction of all three active components. The two tanshinones were obtained in good yields in most NaDES extracts except for ChCl-Ur, in which SAB was also in a low yield (17.96 mg/g). After comparison, L-Pro-Lac was selected as the optimal solvent in the first-round screening, with extraction yields at 42.04 mg/g (SAB), 1.485 mg/g (TIIA) and 0.839 mg/g (CYT).

**Figure 3:**
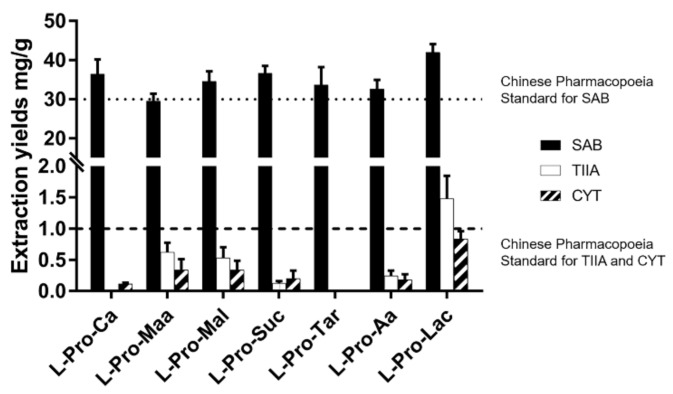
Bioactive components extraction yields from SM using L-Pro-acid type NaDESs (the second-round screening, under the initial conditions).

The second-round screening of NaDESs was then performed, in which L-proline was fixed as HBA and an additional six organic acids besides lactic acid were used as HBDs. As shown in Figure 3 (for detailed data, see Table S3 in Supporting Information), L-Pro-Maa, L-Pro-Mal and L-Pro-Lac showed higher efficiency in the extraction of the three compounds than the others. After all, L-Pro-Lac was still found to be the optimal one, as the extraction yield of TIIA and CYT are much better than the others.

### 2.2. Optimization of the Extraction Factors

**Figure 4:**
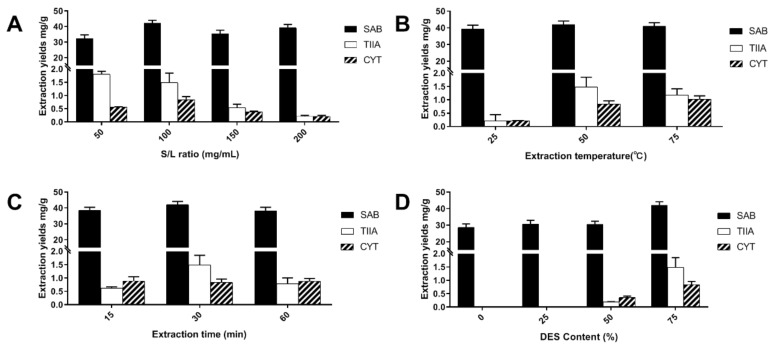
Bioactive components extraction yields from SM using L-Pro-Lac with different parameters: (**A**) S/L ratio (mg/mL); (**B**) Extraction temperature (°C); (**C**) Extraction time (min); (**D**) DES content (%, v/v).

The effect of the extraction factors was further investigated using L-Pro-Lac as a solvent from different aspects. S/L ratio, temperature, time and DES content were studied as key parameters and the results are shown in Figure 4 (for detailed data, see Table S4 in Supporting Information).

The influence of S/L ratio was investigated at 50, 100, 150 and 200 mg/mL while the remaining parameters were kept constant (Figure 4A). As expected, a lower S/L ratio would lead to higher extraction yields of target compounds, but also induce higher costs and waste production of the solvent. The relationship pattern is similar to previous studies on the extraction of medical plants using DES [8,23]. One-hundred milligrams per milliliter (100 mg/mL) was optimized as the suitable value, with the extraction yield at 42.05 (SAB), 1.485 (TIIA) and 0.839 (CYT) mg/g.

Temperature is also an important factor in most extraction processes. It is shown that increased temperature can improve the extraction yield until it reaches a certain level (Figure 4B), which is indicated to be between 50–75 °C in this case. Thus, 50 °C was supposed to be the best extraction temperature as high yields were achieved while the energy cost stayed at a relatively lower level. The effect of extraction time was examined at 15, 30 and 60 min (Figure 4C). The relationship between the yield and time is similar to that of temperature, which suggested 30 min is the most applicable parameter.

Last but not least, the DES content in the extraction solvent mixture was tested at 0, 25, 50, and 75% (v/v) (Figure 4D). The results indicated that increasing DES content would lead to higher yields of the three target compounds, especially for the two tanshinones. The solvent containing 25% L-Pro-Lac and 75% water can hardly extract TIIA and CYT, while the yield of SAB was at 30.69 mg/g. Conversely, the yield of SAB was improved by 37%, and the yields of TIIA CYT also increased dramatically when 75% NaDES was employed in the extraction. The results could reconfirm the good extraction capacities of NaDESs, which exhibited a wide range of polarity tolerance for different compounds.

Finally, the optimal extraction factors are as follows, S/L ratio at 100 mg/g, extraction temperature at 50 °C, extraction time at 30 min, and DES content at 75%. Under the present condition, the yield of SAB reached up to 42.05 mg/g, which is 42% and 53% higher than that of water and methanol; the yields of TIIA and CYT reached up to 1.485 and 0.839 mg/g, which are at the same level as methanol and much better than that of water.

In addition, the recovery of components and solvent was also evaluated [24]. Due to the negligible vapor pressure of NaDES and the high degree of miscibility with water, the separation of active ingredients from NaDES often requires an anti-solvent method, liquid-liquid extraction or microporous resin method [25]. Herein, SAB, TIIA and CYT were recovered with ethyl acetate extraction. The recovery yields were >95% in the first step and decreased to around 70% after the second recycling step. Meanwhile, the recovery yield of NaDES was 94.8% (1st extraction) and 87.8% (second extraction).

### 2.3. Extraction Effect of NaDES for SM from Different Geographic Origins

**Figure 5:**
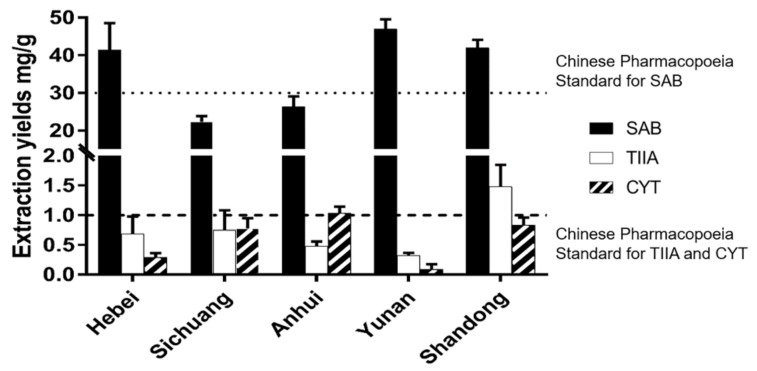
Bioactive components extraction yields from SM from different origins using L-Pro-Lac under the optimal conditions).

Once the optimal extraction parameters were obtained, it was further applied to the extraction of SM samples from different geographic origins. SM samples from both northern and southern China were collected and extracted under the optimal conditions with L-Pro-Lac. As shown in Figure 5 (for detailed data, see Table S5 in Supporting Information), SM samples exhibited diverse contents of the three target compounds. For SAB, the content in SM samples from Hebei, Yunnan and Shandong Provinces was higher than the quality standard in Pharmacopeia of China. For tanshinones, only the SM sample from Yunnan did not reach the pharmacopeia standard, while SM samples from Shandong, Sichuan and Anhui Provinces were above the standard. On the whole, in the present research, SM samples from northern China (Hebei and Shandong) have got higher SAB, TIIA and CYT contents, which are above the pharmacopeia standards (China, US, Europe and Japan). It is noteworthy that the SM sample from Yunnan Province consisted of the highest SAB content but lowest tanshinone contents, which might indicate that it was a good herbal resource for the production of pure SAB product. Most importantly, comparing with the traditional pretreatment process suggested in pharmacopeias, which usually includes two extraction steps by water and ethyl ether respectively, the presented method using NaDES as an extraction solvent significantly reduced the fossil reagent consumption and improved the analysis efficiency. 

### 2.4. DPPH Radical Scavenging Activities of NaDES Extracts

**Figure 6:**
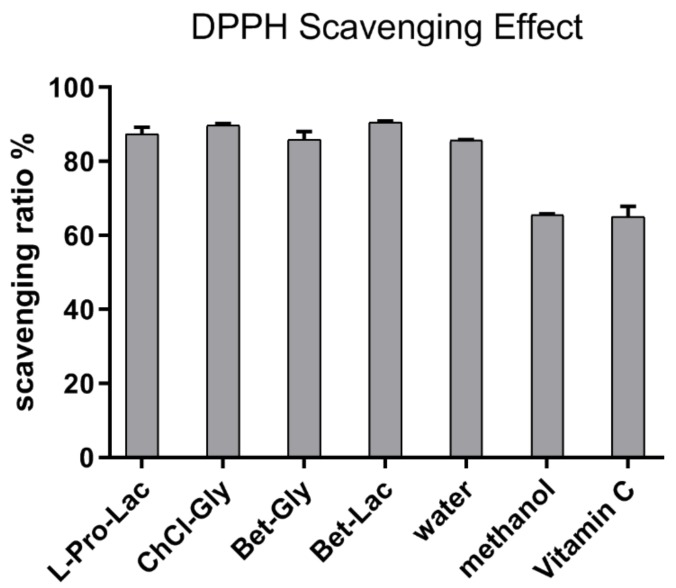
DPPH radical scavenging effect of NaDES and traditional solvent extracts in a concentration of 10 mg SM/mL (0.05 mg/mL vitamin C was used as a positive control).

DPPH is a simplified radical model for antioxidant activity assays, which can give a preliminary evaluation for tested samples. Herein, the capacities of NaDES extracts in DPPH radical scavenging were determined under a well-established protocol. The results are summarized in Figure 6. It could be found that all the selected NaDES extracts of SM showed significant quenching effects toward DPPH radical. The quenching ratio ranges from 85.9% to 90.6% at the concentration of 10 mg SM/mL, which is almost similar with that of water extracts (85.7%) and higher than that of methanol extract (65.6%). As the antioxidative effect of SM was majorly contributed to by SAB, this phenomenon could be explained by the fact that the SAB contents in NaDES extracts are at the same level with that of water extract. The positive control, vitamin C (0.05 mg/mL), can scavenge 65.2% DPPH under the same conditions.

## 3. Discussion

In summary, after two rounds of systematical screening of 22 different NaDESs, the optimal solvent, L-Pro-Lac, was selected to be applied to the extraction of a widely used traditional Chinese medical plant, *Salvia miltiorrhiza*. The results showed that in comparison with traditional solvents (water, methanol, etc.), NaDESs, especially L-Pro-Lac, had significant advantages in the extraction of SAB, TIIA and CYT. After the optimization of extraction parameters, the yields of three targeting compounds were 42.05, 1.485 and 0.839 mg/g, respectively. In addition, the present method was applied to the pretreatment of SM samples from different geographic origins. The results proved it as a more concise and eco-friendly method comparing with pharmacopeia suggested protocols. The DPPH radical scavenging activities of NaDES extracts were also determined in the study to prove the feasibility of NaDES in active component extraction. The application of NaDESs in the extraction of important small molecules from SM is proved to be a green and efficient alternative for traditional solvents. The method might also be expanded to other medical plants with biological interests in the future.

## 4. Materials and Methods 

### 4.1. Materials and Reagents

Dried SM samples were purchased from a local traditional Chinese medicine market (Hangzhou, China) and smashed into powder with a disintegrator. The powder was stored in a dryer until use. Analytical standards, salvianolic acid B (≥99.0%), tanshinone IIA (≥99.0%) and cryptotanshinone (≥98.0%) were all purchased from J&K Scientific Co. Ltd (Beijing, China). Compounds for NaDES preparation, formic acid (88%) and DPPH were all purchased from Shanghai Aladdin Bio-Chem Technology Co. Ltd. (Shanghai, China) with over 95% purity and used directly. HPLC-grade acetonitrile was purchased from Tedia Co. (Fairfield, USA), and the deionized water used in the study was got from a Milli-Q water purification system (Bedford, USA).

### 4.2. Preparation of NaDESs

All NaDESs were prepared following the method in previous reports [10]. Briefly, hydrogen bond acceptors, hydrogen bond donors and water were mixed in different ratios as shown in Table 1. The mixtures were prepared by heating at 80 °C until a homogeneous liquid was formed [26,27]. All NaDESs were kept in a desiccator before usage.

### 4.3. Extraction Procedure

UAE experiments were performed in an ultrasonic bath (KQ5200DE, Kunshan Ultrasonic Instrument Co.). In initial screening, precisely weighted 100.00 mg SM powder was added to 1.00 mL extraction solvent (0.75 mL NaDES and 0.25 mL water) in a 1.5 mL centrifuge tube and vortexed. After extraction under 50 °C, 200 W power for 30 min, the mixture was centrifugated at 16200 g for 10 min. The solution phase was then collected and diluted 10 times with 50% methanol solution for HPLC analysis.

### 4.4. Characterization and Quantification of Extracted Components

HPLC analysis was performed on an Agilent 1200 system including a G1311A QuatPump, a G1322A degasser, a G1315D diode array detector (DAD), a G1329A ALS with a 20 μL loop. In addition, the HPLC column used was Hypersil ODS-C18 (250mm × 4.6mm i.d., 5μm, Agilent). The mobile phase consisted of water containing 0.1% formic acid (phase A) and acetonitrile (phase B). The gradient program was as follows: 0–12 min, 10–22% B, 12–38 min, 22–30% B, 38–50 min, 30–45% B, 50–55 min, 45–90% B, 55–65min, 90% B. The flow rate was 0.8 mL/min, and the wavelengths were 254 nm (for TIIA and CYT) and 280 nm (for SAB).

Calibration curves were established for SAB, TIIA and CYT by plotting the nominal concentrations of standard solutions versus peak areas. The linear ranges, linear regression equations and related details are listed in Table S1 in Supporting Information.

### 4.5. DPPH Radical Scavenging Assay

DPPH radical scavenging activities were evaluated as we have described previously by the use of a microplate [28,29]. In brief, the reaction mixture containing 180 μL DPPH (150 μM freshly made methanol solution) and 20 μL sample/blank solution in methanol was taken in a 96-well microplate and incubated at 37 °C for 30 min. The absorbance was measured at 517 nm. Percentage radical scavenging activity was calculated using the following equation:

*I*(%) = (*A*_blank_ − *A*_sample_)/*A*_blank_ × 100% (1)

where *A*_blank_ is the absorbance of the control reaction mixture without the test samples, and *A*_sample_ is the absorbance of the reaction mixture containing the test samples. The scavenging ratios were expressed as means ± standard deviation for three separate experiments. Vitamin C (0.05 mg/mL) was used as a positive control.

## Figures and Tables

**Table 1:** Different composition of NaDESs studied in this work.

No.	Abbreviation	Type of HBA	Type of HBD	HBA/HBD (Water) Ratio	Appearance at Room Temperature
1	ChCl-Glu	Choline Chloride	D-Glucose	1:1:(2)	Transparent liquid
2	ChCl-Gly		Glycerol	1:2	Transparent liquid
3	ChCl-Lac		Lactic Acid	1:1	Transparent liquid
4	ChCl-Ur		Urea	1:2	Transparent liquid
5	Bet-Glu	Betaine	D-Glucose	1:1:(1)	Transparent liquid
6	Bet-Gly		Glycerol	1:2	Transparent liquid
7	Bet-Lac		Lactic Acid	1:1:(1)	Transparent liquid
8	Bet-Ur		Urea	1:1:(2)	Transparent liquid
9	D-Pro-Glu	D-Proline	D-Glucose	1:1:(5)	Transparent liquid
10	D-Pro-Gly		Glycerol	2:5	Viscous liquid
11	D-Pro-Lac		Lactic Acid	1:1	Viscous liquid
12	D-Pro-Ur		Urea	1:1:(3)	Viscous liquid
13	L-Pro-Glu	L-Proline	D-Glucose	1:1:(5)	Transparent liquid
14	L-Pro-Gly		Glycerol	2:5	Viscous liquid
15	L-Pro-Lac		Lactic Acid	1:1	Viscous liquid
16	L-Pro-Ur		Urea	1:1:(3)	Viscous liquid
17	L-Pro-Ca		Citric Acid	1:1:(2)	Viscous liquid
18	L-Pro-Maa		Malic Acid	1:1:(1)	Viscous liquid
19	L-Pro-Mal		Malonate	1:1:(2)	Viscous liquid
20	L-Pro-Suc		Succinic Acid	1:1:(5)	Viscous liquid
21	L-Pro-Tar		Tartaric Acid	1:1:(5)	Transparent liquid
22	L-Pro-Aa		Ascorbic Acid	1:1:(5)	Transparent liquid

## Supplementary Materials

The following are available online, Table S1. Calibration curves and test ranges for analytes by HPLC, Table S2: Bioactive components extraction yields from SM using NaDESs and traditional solvents, Table S3: Bioactive components extraction yields from SM using L-Pro-acid type NaDESs, Table S4: Bioactive components extraction yields from SM using L-Pro-Lac under different conditions, Table S5: Bioactive components extraction yields from SM from different origins using L-Pro-Lac, Table S6: DPPH radical scanvenging activities of selected SM extracts using NaDESs, Figures S1–S6. Representive HPLC chromatograms of SM extracts.

## Abbreviations

SM	*Salvia miltiorrhiza*
NaDES	natural deep eutectic solvent
SAB	salvianolic acid B
TIIA	tanshinone IIA
CYT	cryptotanshinone
UAE	ultrasonic assisted extraction
