# Supplementary material for: Green and Efficient Ultrasonic-Assisted Extraction of Bioactive Components from Salvia miltiorrhiza by Natural Deep Eutectic Solvents
Source: Molecules. 2019 Dec 29;25(1):140. doi: 10.3390/molecules25010140 (PMC6983008; doi:10.3390/molecules25010140)

Green and Efficient Ultrasonic-Assisted Extraction of Bioactive Components from *Salvia miltiorrhiza* by Natural Deep Eutectic Solvents

Xinping He[#, 1] Jiehong Yang[#,1] Yan Huang,[1] Yin Zhang,[1] Haitong Wan,[1] Chang Li*[1]*

[1] Zhejiang Chinese Medical University, Hangzhou, 310057, PR China

# These authors contributed equally in this work.

* Corresponding Authors

Supporting information

Table of contents | Page

Table of contents	Page
Table S1. Calibration curves and test ranges for analytes by HPLC	2
Table S2. Bioactive components extraction yields from SM using NaDESs and traditional solvents	3
Table S3. Bioactive components extraction yields from SM using L-Pro-acid type NaDESs	4
Table S4. Bioactive components extraction yields from SM using L-Pro-Lac under different conditions	5
Table S5. Bioactive components extraction yields from SM from different origins using L-Pro-Lac	6
Table S6. DPPH radical scanvenging activities of selected SM extracts using NaDESs	7
Figure S1-S6. Representive HPLC chromatograms of SM extracts	8-10

Table S1. Calibration curves and test ranges for analytes by HPLC

No.	Wavelength (nm)	Calibration Curve	R^2	Linear Range (μg · mL^{-1})
SAB	280	Y=14.226X-349.59	0.9997	100.00-500.00
TIIA	254	Y=82.605X+42.706	0.9995	1.250-30.000
CYT	254	Y=94.560X+51.3	0.9997	1.250-20.000

Table S2. Bioactive components extraction yields from SM using NaDESs and traditional solvents (the first-round screening, Figure 2, values are expressed as mean ± SD)

Slovent	SAB (mg/g)	TIIA (mg/g)	CYT (mg/g)
ChCl-Glu	33.980±2.038	0.435±0.094	0.312±0.012
ChCl-Gly	41.830±2.639	0.649±0.064	0.373±0.103
ChCl-Lac	35.450±1.032	0.125±0.084	0.340±0.013
ChCl-Ur	17.960±1.347	<0.125	<0.125
Bet-Glu	38.030±2.584	0.945±0.024	0.433±0.122
Bet-Gly	39.660±3.238	1.045±0.146	0.530±0.127
Bet-Lac	39.610±1.674	0.268±0.003	0.284±0.037
Bet-Ur	32.490±1.367	0.301±0.019	0.168±0.028
L-Pro-Glu	37.670±2.221	0.569±0.025	0.227±0.014
L-Pro-Gly	38.730±1.034	1.307±0.145	0.613±0.131
L-Pro-Lac	42.035±2.070	1.485±0.361	0.839±0.121
L-Pro-Ur	32.060±2.383	0.332±0.026	0.222±0.002
D-Pro-Glu	39.420±2.063	0.695±0.128	0.264±0.003
D-Pro-Gly	34.780±3.248	1.049±0.004	0.507±0.028
D-Pro-Lac	33.930±1.783	0.873±0.038	0.568±0.023
D-Pro-Ur	38.740±3.285	0.428±0.032	0.285±0.015
water	28.782±1.284	<0.125	<0.125
methanol	19.665±1.872	1.788±0.036	0.761±0.126

Table S3. Bioactive components extraction yields from SM using L-Pro-acid type NaDESs (the second-round screening, Figure 3, values are expressed as mean ± SD)

Slovent	SAB (mg/g)	TIIA (mg/g)	CYT (mg/g)
L-Pro-Ca	36.424±3.754	<0.125	<0.125
L-Pro-Maa	29.523±1.920	0.625±0.150	0.342±0.170
L-Pro-Mal	34.608±2.510	0.535±0.171	0.340±0.147
L-Pro-Suc	36.658±1.843	0.129±0.030	0.203±0.127
L-Pro-Tar	33.705±4.525	<0.125	<0.125
L-Pro-Aa	32.621±2.331	0.247±0.082	0.186±0.085
L-Pro-Lac	42.035±2.070	1.484±0.361	0.839±0.121

Table S4. Bioactive components extraction yields from SM using L-Pro-Lac under different conditions (Figure 4, values are expressed as mean ± SD)

Extraction Condition		SAB (mg/g)	TIIA (mg/g)	CYT (mg/g)
A. S/L Ratio (mg/mL)	50	32.290±2.392	1.801±0.113	0.567±0.013
	100	42.035±2.070	1.484±0.361	0.839±0.121
	150	35.360±2.308	0.539±0.136	0.388±0.023
	200	39.320±2.113	0.218±0.032	0.217±0.031
B. Extraction Temperature(℃)	25	33.320±2.308	0.218±0.227	0.217±0.011
	50	42.035±2.070	1.484±0.361	0.839±0.121
	75	41.010±2.112	1.184±0.228	1.013±0.139
C. Extraction Time (min)	15	38.460±1.952	0.620±0.053	0.893±0.153
	30	42.035±2.070	1.484±0.361	0.839±0.121
	60	38.050±2.372	0.780±0.224	0.878±0.104
D. DES Content (%)	0	28.780±1.985	<0.125	<0.125
	25	30.690±2.219	<0.125	<0.125
	50	30.540±1.893	0.196±0.006	0.353±0.056
	75	42.035±2.070	1.484±0.361	0.839±0.121

Table S5. Bioactive components extraction yields from SM from different origins using NaDES-15 (L-Pro-Lac, Figure 5, values are expressed as mean±SD)

SM origin	SAB (mg/g)	TIIA (mg/g)	CYT (mg/g)
Hebei	41.424±7.130	0.690±0.288	0.291±0.073
Sichuang	22.334±1.491	0.751±0.333	0.778±0.173
Anhui	26.336±2.724	0.482±0.073	1.037±0.107
Yunnan	46.988±2.579	0.326±0.040	0.093±0.081
Shandong	42.035±2.070	1.485±0.361	0.839±0.121

Table S6. DPPH radical scanvenging activities of selected SM extracts using NaDESs

Slovents	DPPH Scavenging Effect(%) Mean±SD
L-Pro-Lac	87.481±1.775
ChCl-Gly	89.773±0.504
Bet-Gly	85.942±2.169
Bet-Lac	90.612±0.359
water	85.720±0.178
methanol	65.625±0.243
Vitamin C (0.05 mg/mL)	65.154±2.761

Figure S1-S6. Representive HPLC chromatograms of SM extracts

Figure S1. HPLC chromatograms of SM extracts using water (wavelength=280 nm)

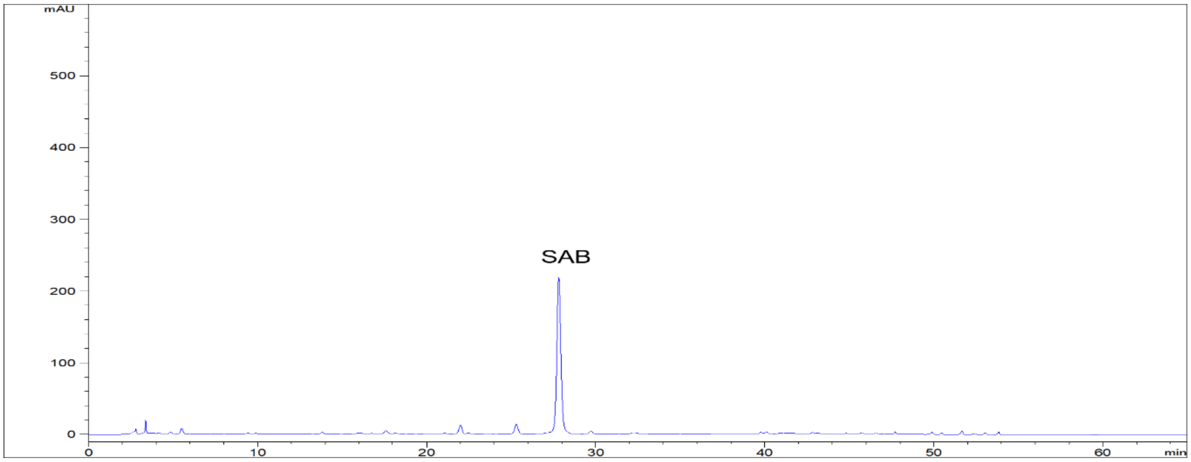

Figure S2. HPLC chromatograms of SM extracts using methanol (wavelength=280 nm)

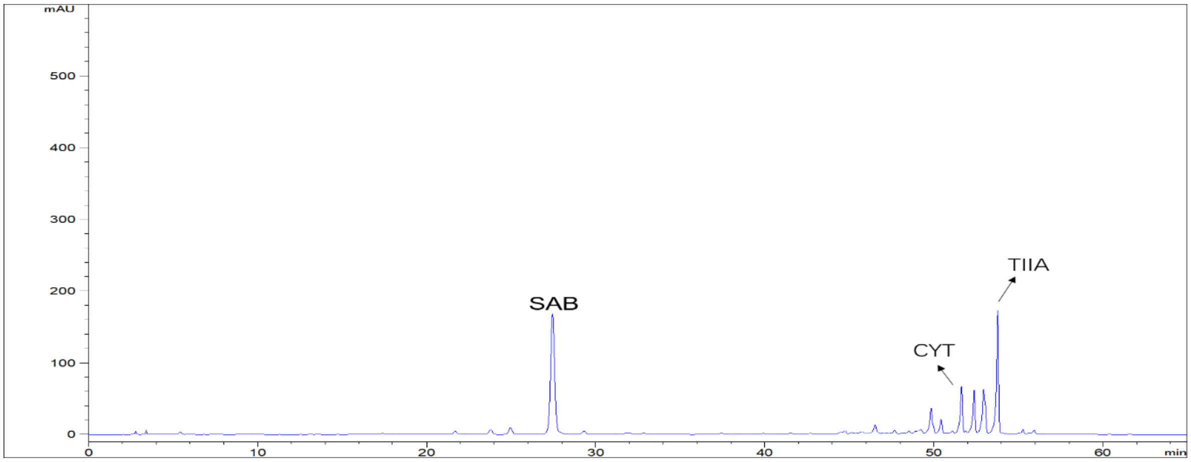

Figure S3. HPLC chromatograms of SM extracts using NaDES-2 (ChCl-Gly, wavelength=280 nm)

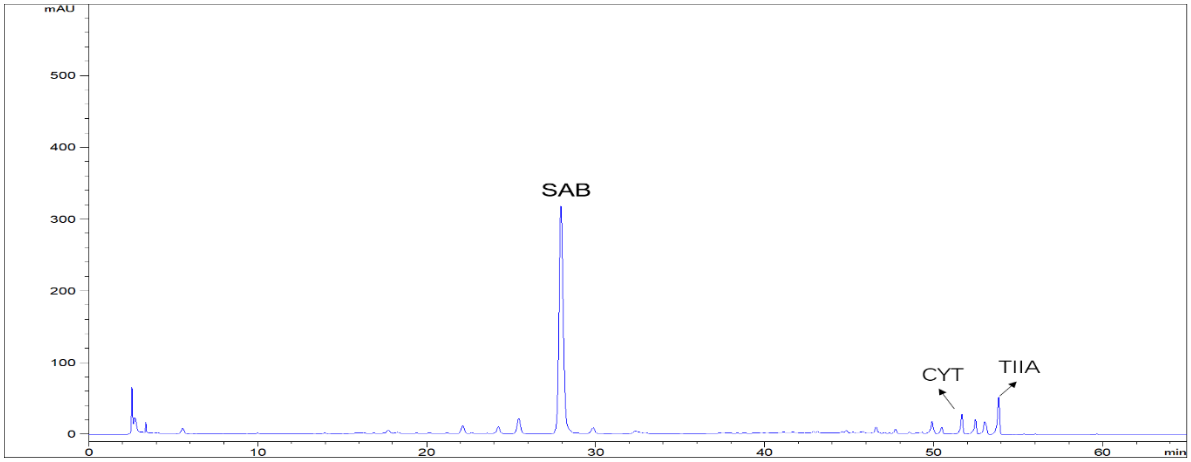

Figure S4. HPLC chromatograms of SM extracts using NaDES-6 (Bet-Gly, wavelength=280 nm)

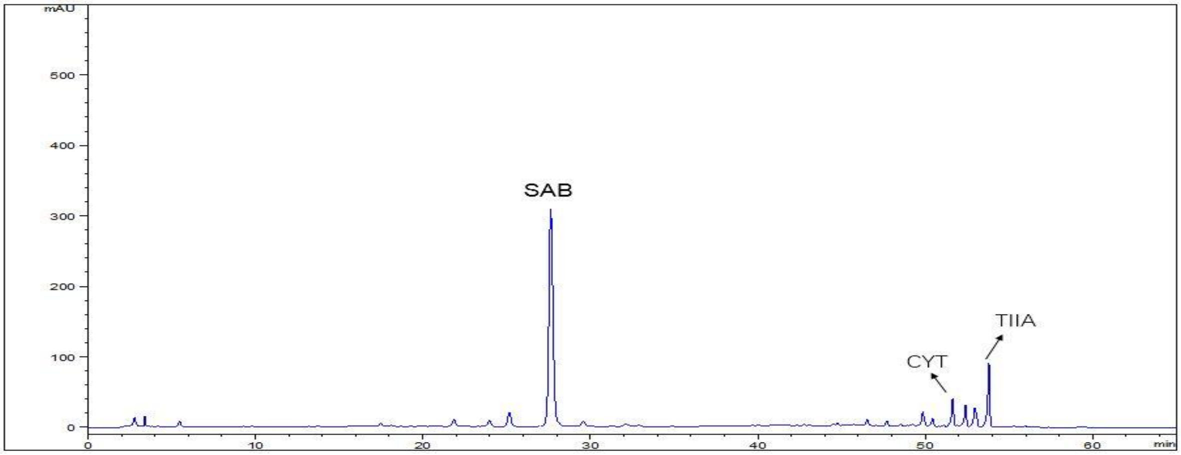

Figure S5. HPLC chromatograms of SM extracts using NaDES-7 (Bet-Lac, wavelength=280 nm)

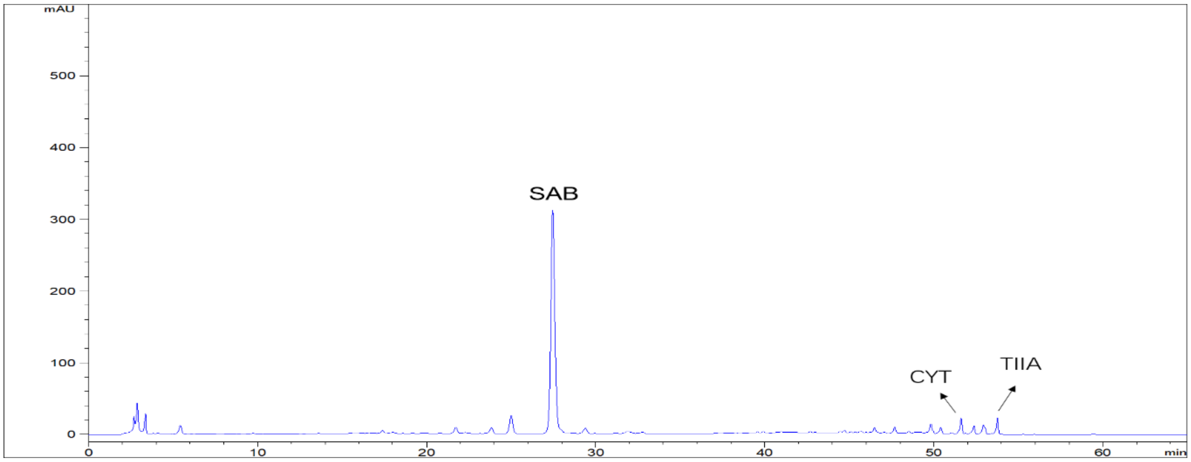

Figure S6. HPLC chromatograms of SM extracts using NaDES-15 (L-Pro-Lac, wavelength=280 nm)

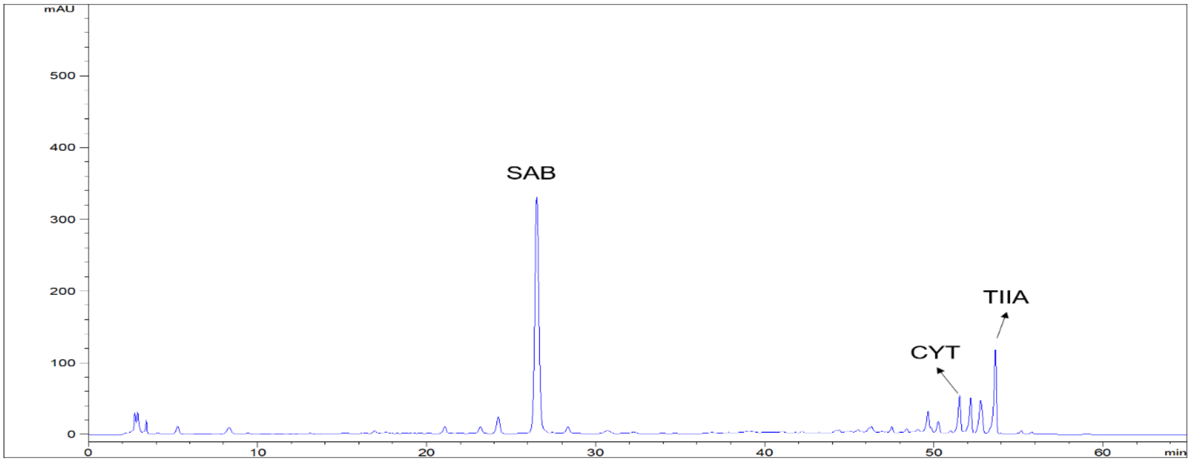

Supplement: Supplementary file 1 [file molecules-25-00140-s001.pdf]
